# Prognosis1Read before the Annual Meeting of the Pennsylvania State Veterinary Medical Association, March, 1899.

**Published:** 1899-07

**Authors:** J. C. Michener

**Affiliations:** Colmar, Pa.


### PROGNOSIS.[1]

By J. C. MICHENER, V.S.,
COLMAR, PA.

THE elements of success in the practice of our profession are threefold :

First and most important is actual knowledge pertaining to all branches of the art; second, executive ability, or the faculty of applying knowledge; third, prognosis, or the ability to forecast the results of the diseases and conditions met in practice.

Skill and discretion in the use of the tongue is largely the making of a practitioner. We must talk. Our patients are property, and aside from the attachment the owner has for his faithful animals our services become a matter of dollars and cents. To experienced practitioners I need not portray the close questioning to which we are subjected, and scarce need tell that the client's estimate of us will be determined by the verification of our answers.

To the young man I would say, the safest plan (for both old and young) is to prognosticate as little as possible.

Direct questions must be answered, or we will be in the position of the simple boy whose mother had ordered him to keep his mouth shut on a particular occasion. Being questioned, and making no reply, one guest remarked to another, "I

[1] Read before the Annual Meeting of the Pennsylvania State Veterinary Medical Association, March, 1899.

believe the boy is simple," when he blubbered out, "Mother! mother! they found me out, and I did not say a word."

Much skill can be exercised in making provision for the unforeseen circumstances that may arise. If the lame horse does not strain himself over again, the sick one take fresh cold, the surgical case contract blood-poisoning or develop lockjaw, we can hope for favorable results. "Look wise, bu say little." Grave looks, a shake of the head, upon taking the pulse or temperature, frequent visits, anxious inquiry when the attendant is seen before the patient, all tend to make it a serious case, assuage grief when terminating fatally, and augmenting the rejoicing when the case recovers.

I am talking particularly to the young men, and can assure them that these little artifices are justifiable in self-protection under the difficult circumstances.

The human practitioner's powers of forecast are not so severely tested. He is not required to answer so definitely; simply, is it a serious case, giving him a chance to err upon the safe side. But the veterinarian is asked bluntly, Can you pull him through? Which must be answered by a yes or no, or a hopeful or doubtful. If no, or doubtful, your services are discredited and likely discarded at once. If an affirmative answer is followed by a negative result, you are branded a humbug.

How is the practitioner to prepare himself best for this difficult part of his work? A close study of the natural course of diseases, their period of duration, the weakness or idiosyncrasy of the patient and the hygienic conditions under which he must be treated, together with the history, the stage in which he is found, the extent of pathological change, all of which, coupled with great discretion, will enable the veterinarian to answer the unavoidable questions in such a way as to convince his patron that he understands his business.

Not only this, but the knowledge just enumerated is essential to avoid being robbed of the fruits of your skill and care. Many funny things occur in practice. Dr. Brown has been treating a spavin for several weeks. The benefits are not apparent to the owner, and he is dismissed and Jones employed. Anchylosis is about being completed; Jones has the toe shortened, gives a little judicious exercise and monkey treatment. Lo! the horse soon goes sound, and Jones scores a mighty victory. Brown has his revenge. Jones has treated a case of strangles until the suppurative stage is near, gets the bounce, and Brown shows them how to clean them out. He is usually the lucky man who is called near the close of an endemic. The cases are less severe and show his superior treatment. Typhoid influenza and parturient apoplexy often give a man much unearned glory.

We are frequently asked for an opinion upon some abnormal condition, especially when the animal is about being sold. Does or will it disqualify the animal? Other opinions will be freely proffered and sought after, and your prognosis, in the case, surely put to the test. Many cases will not admit of a positive prediction, and many reputations have been shattered by making positive assertions. Still worse when a man is off in his diagnosis, pronouncing a case of bruised or punctured frog, severe hip- or shoulder-lameness; a broken tooth or foreign body fast in the mouth, a case of choking distemper; and there are lots of similar mistakes that amount almost to professional suicide.

